# Whose game is it anyway? Palworld and the new frontier of intellectual property in eSports

**DOI:** 10.3389/fspor.2025.1652617

**Published:** 2025-11-27

**Authors:** Iván Vargas-Chaves, Yannina Inoñán-Mujica, Jesús Manuel González-Herrera, Julissa Sharai Anacleto-Gómez

**Affiliations:** School of Law, Universidad Señor de Sipán, Pimentel, Perú

**Keywords:** eSports, intellectual property, copyright, contracts, trade dress

## Abstract

This paper explores key concepts of intellectual property in shaping the legal framework and challenges of the burgeoning eSports industry. The author argues that intellectual property extends beyond mere asset protection, acting as a strategic factor that drives competitiveness, innovation, and collaboration within this dynamic ecosystem. Furthermore, through a theoretical framework, the analysis of the potential legal issues surrounding the Palworld case as an illustration of tensions between creativity and rights, the examination of contracts and copyright licenses, and a proposal of guidelines against infringements, the paper highlights the need for a comprehensive approach to intellectual property in eSports. The findings underscore the importance of protecting copyrights, managing contracts and licenses, and safeguarding trademarks and Trade Dress to ensure the sustainable development and integrity of the industry.

## 1 Introduction

The eSports industry has emerged as a significant global phenomenon, characterized by the convergence of video game competitiveness, dynamic digital content creation, and the appeal of live entertainment. This trend has been noted in the literature (1, 2). This exponential growth has generated a complex ecosystem where the protection of intellectual property has become fundamental for the sustainability and long-term development of eSports organizations (3).

In this context, the present article addresses the following research question: What is the role of intellectual property in shaping the legal framework and the dynamics of infringement, copying, and imitation in the eSports industry? It is hypothesized that intellectual property not only constitutes a set of legal tools for asset protection but also a strategic factor that influences competitiveness, innovation, and collaboration within the eSports ecosystem.

Consequently, the general objective is to analyze the importance of intellectual property in the eSports industry, examining the various forms in which it manifests and the specific challenges it poses. To achieve this objective, the article is structured as follows: First, a theoretical framework on the intersection between intellectual property and eSports is presented.

Subsequently, the Palworld case is analyzed as an illustrative example of the tensions between creative inspiration and intellectual property rights. Following this, the role of copyright contracts and license agreements is examined as key instruments for regulating the use and distribution of creative assets in eSports. Finally, a proposal for guidelines is presented to address copyright infringements in the digital environment of eSports.

The main findings of the article highlight the need for a comprehensive approach to intellectual property in eSports. This approach should encompass the protection of copyrights, the management of contracts and licenses, and the safeguarding of trademarks and Trade Dress. It is concluded that intellectual property is not only a mechanism for legal protection but also a key factor for the sustainable development and integrity of the eSports industry.

## 2 The eSports industry and legal complexity

The eSports industry has emerged as a global phenomenon of great magnitude, integrating the competitiveness of video games, the dynamic creation of digital content, and the appeal of live entertainment (4, 5). This constantly expanding ecosystem generates significant revenue and attracts a massive worldwide audience. This growth is substantial (6).

However, behind the spectacle of tournaments and broadcasts, eSports organizations face an intricate legal landscape, where the comprehensive protection of intellectual property becomes a key aspect for their sustainability and long-term growth (7). During their activities, eSports teams and leagues are immersed in various legal issues related to intellectual property. These multifaceted legal dimensions have been explored by several authors (8–11).

Firstly, securing broadcasting rights for tournaments allows for the dissemination of competitions across different platforms, generating income through sponsorships, advertising, and subscriptions. Therefore, ensuring ownership or appropriate licenses for rebroadcasting is essential to avoid infringements and legal disputes.

Secondly, the creation of original content represents another area of interest, stemming from the so-called “arena right” (12). Teams and players generate a wide variety of materials, including music, comics, and distinctive branding. Safeguarding these intangible assets through copyright and trademarks is indispensable to preserve their value and prevent unauthorized use by third parties (13).

Guaranteeing intellectual property protection is not only a legal obligation but also an intelligent business strategy. It allows organizations to control the exploitation of their assets, maximizing their economic potential (14, 15). Furthermore, it strengthens their identity and reputation in an increasingly competitive market (16). Thus, investing in legal protection strategies contributes to the stability and sustainable growth of the industry.

Currently, the business model of eSports organizations is based on the prolific creation of digital content, brand development, and constant interaction with a global audience (10, 11, 17, 18). This dynamic context requires organizations to implement a comprehensive strategy to ensure the protection of creative resources generated and used on various eSports platforms. This is achieved through measures aimed at safeguarding intellectual property rights and fostering a collaborative environment.

## 3 Esports and intellectual property

The multifaceted nature of operations in eSports demands a copyright protection approach that extends beyond traditional measures (19). This is due to the constant generation of original content, including streams, videos, graphics, music, and narratives, which require robust protection to prevent unauthorized reproduction and distribution (20).

Intellectual property law functions as the primary normative architecture within the eSports ecosystem. It establishes the foundational rules of engagement, delineating the boundaries of permissible competitive behavior (21, 22). This legal framework does not merely protect static assets; it actively shapes the strategic decisions of developers, teams, and platforms, defining what is considered a legitimate market offering vs. an unlawful encroachment.

The economic theory underpinning intellectual property is particularly salient in eSports. Copyright and trademark protections provide the necessary incentives for the substantial investment required to develop, market, and sustain a competitive gaming title (23). Without a predictable legal framework to ensure a return on this creative investment, the impetus for innovation would be severely diminished, leading to a market dominated by imitation rather than novel creation.

A central theoretical tension arises in distinguishing between creative homage and actionable infringement. The eSports industry, like other creative fields, thrives on iteration and the evolution of established genres. Intellectual property doctrine must therefore navigate a delicate balance, fostering an environment where developers can draw inspiration from existing works while simultaneously preventing slavish copying or imitation that misleads consumers and usurps market share.

Beyond its purely protective function, intellectual property operates as a strategic tool that directly influences market dynamics. Rights holders can leverage their portfolios offensively to create barriers to entry or challenge competitors, as seen in patent and trade dress disputes. Defensively, a robust IP strategy is essential for securing partnerships, attracting investment, and maintaining brand integrity in a saturated and visually-driven marketplace (24, 25).

The legal framework of eSports is increasingly shaped by para-regulatory forces, namely the digital platforms themselves. The terms of service and automated enforcement systems of platforms like Twitch and YouTube function as a secondary layer of governance. These private rulesets often interpret and apply intellectual property principles—such as fair use and takedown notices—with an immediacy that can shape the dynamics of content creation and imitation more rapidly than traditional jurisprudence.

Finally, the “dynamics of imitation” are not governed solely by formal law. A complex interplay exists between codified intellectual property rights and the informal, socially-enforced norms of the fan community. Activities such as modding, fan art, and community-run tournaments often operate in a legal gray area. How rights holders choose to enforce—or strategically ignore—these activities profoundly shapes the collaborative and cultural health of their ecosystem.

Likewise, developing a distinctive brand through logos, team names, and slogans demands the use of trademarks, which complement copyrights in protecting visual and identity elements (26). Moreover, active interaction with the audience involves managing user-generated content and safeguarding the organization's own content shared in these spaces. These user-interaction dynamics have been explored (27). Consequently, intellectual property management in eSports must consider both the protection of primary content and the management of rights related to public interaction.

To ensure effective protection, eSports organizations have been implementing a guiding strategy that comprises several key components, from clear internal policies to contracts specifying intellectual property responsibilities.

Technical protection measures have also gained prominence, such as watermarks, digital rights management (DRM) systems, and monitoring of online platforms to detect infringements (28). Concurrently, organizations have worked on formalizing solid contractual agreements with creators, broadcast partners, and sponsors, which is essential for defining ownership and usage rights (29).

The inclusion of specific clauses on copyright in licensing, assignment, and collaboration contracts becomes a key tool for preventing conflicts and ensuring the protection of the organization's interests (30–32). The premise is that contracts are the foundation of the intellectual property protection chain, as electronic games are creative works protected by copyright. This protection extends to source code and diverse artistic elements like characters, settings, and music (33, 34).

In this context, the effective management of intellectual property represents a fundamental pillar for the stability and growth of the eSports industry. Copyright license agreements are essential contractual tools to regulate the use and distribution of creative assets (35). It is imperative that eSports organizations ensure all involved professionals formalize detailed contracts with explicit copyright clauses (36–38).

These agreements must unequivocally define the ownership of the generated content, whether it is a match video, a musical track, or promotional material (39). This clearly establishes who holds the rights and has the authority to control the work's use and exploitation. Furthermore, license agreements delineate the specific scope of the rights granted to the different parties involved.

For example, an agreement with a streamer can specify the terms for using the team's brand, while a contract with a content creator may detail rights for music use in a promotional video. These agreements also regulate rights for sponsors and commercial partners (40). Contracts establish limits on how these entities can use copyrighted content, preventing unauthorized monetization and ensuring use conforms to agreed-upon terms.

Another relevant aspect is their function in ensuring compliance with the regulations of digital platforms. Contracts often require streamers and influencers to abide by the copyright policies of platforms like Twitch or YouTube (41). This fosters a culture of respect for intellectual property and mitigates infringement risks that could lead to sanctions.

In sum, copyright license agreements are the foundation of legal protection in eSports, but their importance transcends mere asset safeguarding. The legal clarity they provide prevents potential conflicts and strengthens professional relationships by establishing a framework of mutual understanding and respect for rights.

## 4 The “Palworld” case

The term eSport refers to any competitive game played on an electronic device. This broad category includes PC and console games like Fortnite or League of Legends, as well as mobile and web-based games. Palworld fits within this framework as a competitive, open-world survival and adventure game on platforms like Windows, Xbox, and PlayStation 5.

Initially announced in 2021, the Japanese company Pocket Pair launched the game in January 2024. The game introduces players to a world inhabited by “Pals”, which can be captured for construction, exploration, and combat; they can even be equipped with firearms. Player interaction occurs through digital interfaces, where decisions and actions shape the game's development.

A significant portion of its initial success was attributed to its engaging gameplay. However, what truly propelled Palworld to notoriety was its pronounced similarity to the iconic Pokémon franchise. The resemblances in the design of the “Pals”, capture mechanics, and overall aesthetic led many to label it a high-profile “Pokémon clone”, generating intense debate about creative inspiration and intellectual property infringement (42).

The game's narrative places users in a universe populated by Pals, which players can fight and capture for various activities. The storyline allows players to equip Pals with firearms, leading to the nickname “Pokémon with guns”. The game also features the option to use creatures as a food source or assign them to labor in mines and factories.

Overall, Palworld has been well-received for its intuitive gameplay, extensive content, and high-quality graphics. Subsequently, it sparked considerable debate due to its similarities with the Pokémon series, particularly in creature design and game mechanics (43). This situation has revived a critical discussion concerning creative inspiration and potential intellectual property violations in eSports.

eSports constitute complex works integrating multiple elements protected by intellectual property, including code, character designs, music, and trademarks. Despite criticism, Palworld captured the attention of millions, distinguishing itself through a unique blend of survival, exploration, and creature capture. Its rapid ascent and controversy have established it as a noteworthy phenomenon.

The legal action initiated by Nintendo and The Pokémon Company against Pocketpair, Inc. in the Tokyo District Court highlights a critical need for legal precision (44). The plaintiffs’ public statements reference claims of patent infringement alongside demands for compensation for damages to their commercial image, which aligns with the concept of “Trade Dress”.

In addition, it is important to distinguish these as separate legal claims operating under different frameworks. Patent law protects novel inventions and functional mechanics, whereas trade dress—a concept rooted in trademark and unfair competition law—protects the total image and overall aesthetic “look and feel” of a product that identifies its source.

While the lawsuit may proceed on multiple fronts, the core of the public controversy regarding visual similarities falls squarely within the domain of unfair competition and the protection of a product's distinctive appearance, which in Japan is primarily governed by the Unfair Competition Prevention Act, rather than a direct analogue to U.S. trade dress doctrine.

## 5 Case analysis and lessons learned

Given that the litigation between Nintendo and Pocket Pair is ongoing, any analysis of its merits must remain speculative. The legal questions at its core are multifaceted, touching upon patent law, copyright, and trade dress. A definitive outcome is not yet known, and thus, this section will explore the potential legal pathways and doctrinal challenges the case presents. The analysis will proceed hypothetically, examining arguments that either party might advance and considering how established legal principles could be applied. For instance, if the court determines that certain game mechanics are protected by valid patents, the inquiry would shift to whether *Palworld*'s features fall within the scope of those patent claims.

The legal problem in the Palworld case is whether it constitutes an intellectual property infringement by imitating the aesthetic of the Pokémon franchise. This case highlights the complex balance between artistic inspiration and IP rights, bringing the debate to the concept of Trade Dress, which is defined as the total image and overall impression of a product, including elements like design, color, and shape that identify with the consumer.

The Palworld controversy serves as a critical case study for interrogating this theoretical framework in practice. It moves the research question from the abstract to the concrete, providing a lens to analyze how the legal framework is actively being shaped and contested. The case exemplifies the inherent tension between the unprotectable “idea” of a monster-taming genre and the protectable “expression” of its specific aesthetic elements.

This situation thus illustrates the “dynamics of imitation” at the heart of the research question. The strategic decision by a developer to adopt an aesthetic closely resembling a market leader challenges the legal framework's ability to police the boundary between homage and appropriation (45). It forces a theoretical and judicial reckoning with how much “feel” a competitor can permissibly borrow before engaging in unlawful imitation or unfair competition.

To resolve this tension, the theoretical framework must pivot to a doctrine specifically designed to govern imitation of a product's overall image. This concept is central to understanding how intellectual property shapes the eSports environment beyond simple copyright. It is the primary legal tool for analyzing the dynamics of imitation when the alleged copying is not of code, but of the holistic “look and feel” that defines a product's identity (45).

In United States jurisprudence, the protection of trade dress is contingent upon two primary factors: distinctiveness and the likelihood of consumer confusion. Distinctiveness can be either inherent—where a product's design is so unique that it is immediately identifiable with its source—or it can acquire a “secondary meaning” over time, where consumers come to associate a specific look and feel with a particular brand. For a franchise as globally recognized as Pokémon, establishing secondary meaning would likely be a straightforward evidentiary matter. The more contentious issue would be demonstrating that *Palworld*'s overall aesthetic is similar enough to cause a likelihood of confusion among consumers as to the source or affiliation of the game.

This doctrine's relevance is particularly amplified within the digital ecosystem that eSports inhabit. Unlike tangible goods, a video game downloaded from a digital storefront or viewed on a streaming platform lacks traditional physical packaging. Consequently, the game's “look and feel”—its artistic style, user interface design, character models, and overall aesthetic—ceases to be merely ornamental and becomes the primary, and often sole, signifier of the product's source at the point of consumer contact. This digital trade dress effectively *is* the packaging. Its role in brand identification is therefore paramount, making the “likelihood of confusion” standard critically important for navigating a saturated, visually-driven marketplace where a consumer's purchasing decision relies heavily on this immediate visual presentation.

This legal framework becomes more complex in a transnational context, which is inherent to the eSports industry. While the U.S. concept of trade dress is influential, its application varies internationally. For example, some jurisdictions may rely more heavily on unfair competition laws or registered design rights rather than a common law concept of trade dress. The digital and borderless nature of video games means that an alleged infringement occurs simultaneously in multiple legal territories, creating a jurisdictional challenge. Consequently, a legal strategy must account for these international variations, as the standards for proving distinctiveness and consumer confusion may differ significantly from one country to another.

To further contextualize this jurisdictional complexity, it is useful to compare the U.S. approach with other key legal regimes. In the European Union, for instance, protection for a game's “look and feel” may be sought through a combination of avenues, including claims under unfair competition laws of member states, passing off actions in common law jurisdictions, and potentially through unregistered community design rights, which protect the appearance of a product for a limited period. In Japan, the legal battleground is different yet again.

The Unfair Competition Prevention Act provides the primary legal basis for claims against the imitation of a product's configuration or overall presentation. Rather than focusing on “likelihood of confusion” in the same manner as U.S. trademark law, the cited Act's relevant provisions often center on the unauthorized use of a well-known indication of goods or business, making the establishment of Pokémon's fame a critical, though likely straightforward, element of the case.

Beyond jurisdictional variances, applying trade dress doctrine to video games requires a nuanced analysis of what constitutes a protectable “total image”. A significant hurdle is the functionality doctrine, which dictates that functional elements of a product—such as game mechanics or rules of play—cannot be protected as trade dress, as this would improperly stifle competition. Courts must therefore distinguish between purely aesthetic, non-functional elements that serve to identify the source (e.g., unique character art styles, color palettes, user interface design) and functional aspects that are essential to the game's operation.

Furthermore, establishing consumer confusion in a digital context presents unique evidentiary challenges. The analysis would likely involve examining not only the games themselves but also their presentation in streamed environments on platforms like Twitch and YouTube, where the overall context of the broadcast could either mitigate or exacerbate the likelihood of confusion among viewers.

In eSports, this concept manifests in team logos, color palettes, uniform design, and broadcast presentation. The jurisprudence surrounding Trade Dress in the digital realm is still developing. Courts will face the challenge of applying traditional legal concepts to a virtual environment, analyzing factors like distinctiveness and the likelihood of consumer confusion.

The central tension in the *Palworld* case highlights a foundational issue in intellectual property law: the threshold separating permissible creative inspiration from unlawful infringement. This distinction often hinges on the idea-expression dichotomy, a legal doctrine asserting that copyright protects the specific expression of an idea, but not the idea itself. In the context of video games, elements such as a “monster-taming” genre or open-world survival mechanics would likely be considered unprotectable ideas. However, the specific artistic rendering of characters, their unique combination of features, and their overall presentation may constitute protectable expression.

Comparative jurisprudence from prior video game disputes offers valuable context for this distinction. For instance, the series of lawsuits against Epic Games concerning *Fortnite*'s dance emotes (e.g., *Pellegrino v. Epic Games, Inc.*) illustrates the difficulty of protecting discrete elements under copyright. The courts generally found that individual dance steps or simple routines lacked the requisite originality and authorship to qualify for copyright protection, thereby falling into the realm of permissible inspiration or unprotectable ideas. These cases underscore that not every creative element within a game is legally defensible, particularly when those elements are generic or common in a particular genre.

A more direct parallel can be found in the litigation between *PlayerUnknown's Battlegrounds* (PUBG) and *Fortnite*. PUBG Corporation sued Epic Games, alleging that *Fortnite*'s “Battle Royale” mode copied substantial elements of its game, including the game's structure and overall feel. Although the lawsuit was later withdrawn, it centered on the copyrightability of a game's genre and mechanics. This dispute brought the *scènes à faire* doctrine to the forefront—the principle that elements standard or indispensable to a particular genre (such as a shrinking play area in a battle royale game) are not protected by copyright. This precedent is highly relevant, as a potential defense for *Palworld* would be to argue that the elements it shares with Pokémon are generic conventions of the monster-taming genre.

While the specific reasons for the withdrawal were not publicly disclosed, it is broadly understood to have concluded in a confidential settlement. This outcome is itself instructive, highlighting the immense costs and significant risks inherent in pursuing such foundational copyright claims.

Proceeding to a definitive judgment carried the substantial risk of an adverse precedent—a court formally ruling that the battle royale structure and its constituent mechanics are, indeed, unprotectable *scènes à faire*. Such a ruling could have ultimately proven more damaging to the plaintiff's long-term strategic interests than the alleged infringement itself, illustrating how the limitations of litigation are often defined by commercial pragmatism and risk aversion rather than the perceived strength of the legal argument alone.

In addition to legal protection, organizations use branding and marketing to strengthen their Trade Dress. Investing in high-quality design, ensuring visual consistency, and maintaining community interaction can consolidate a distinctive image and deter imitation (16).

Should the case proceed to a substantive analysis, the court's decision will likely depend on a granular comparison of the assets in question. If the tribunal were to find substantial similarity in the expression of the character designs, Pocket Pair's defense might pivot to arguing transformative use or parody. They could contend that the introduction of firearms and darker thematic elements, such as labor and consumption of the “Pals”, transforms the original work's meaning and purpose, thereby constituting a fair use or a legally distinct creation. Such a defense, however, would require a nuanced evaluation of whether the new work primarily serves as a market substitute for the original or if it genuinely offers a new, critical, or creative perspective.

The Palworld case shows the need for legal teams in eSports to fully understand the regulatory framework of intellectual property. It is imperative that all stakeholders, from developers to content creators, possess solid knowledge of its scope. Another lesson is the necessity of well-defined license agreements to mitigate potential infringement problems, especially in fan-organized events.

Developers must ensure licenses for copyrighted elements are clearly established. This involves creating transparent terms of use and being willing to grant specific licenses for non-commercial uses that enrich the competitive scene. The Palworld case serves as a wake-up call to adopt a culture of respect for intellectual property and implement due diligence in content creation.

Clarity in agreements not only protects developers' rights but also empowers organizers and content creators, fostering a collaborative ecosystem. It is essential to internalize the potential for legal action and the severe consequences of unauthorized use, as the damage to a company's reputation can be irreparable.

## 6 A proposed approach: guidelines for addressing intellectual property infringements in eSports

The eSports industry operates on digital platforms like Twitch and YouTube, where protecting copyrights is a complex but important task. As Zhou (46) points out, eSports organizations rely on automated tools offered by these platforms to address infringements. These platforms have developed sophisticated content identification systems that compare user-uploaded content against databases of protected works.

In addition to proactive identification, takedown procedures under the Digital Millennium Copyright Act are a fundamental reactive mechanism (47–49). This allows copyright holders to notify platforms of unauthorized content and request its prompt removal. Efficiency of these processes is essential for minimizing the impact of infringements (50).

Digital platforms also offer monitoring tools that enable organizations to track their content across services (51). This facilitates a more rapid and effective response to copyright violations, allowing legal teams to act on early detection (52). Close collaboration with platforms like Twitch and YouTube is a determining factor in protecting copyrights in eSports.

This synergy ensures organizations can act immediately when improper use of their material is identified, expediting notification and removal processes (53, 41). Regarding dispute resolution, teams, developers, and creators need an impartial forum for conflicts that span multiple jurisdictions. This need arises from the cross-border nature of eSports.

These infringements are often addressed through the World Intellectual Property Organization's Arbitration and Mediation Center, which offers mediation, arbitration, and expert determination (54, 55). This body handles diverse copyright disputes, such as controversies over game plots, unauthorized rebroadcasting, and royalty payment disagreements. This highlights the relevance of alternative dispute resolution for the unique legal challenges in this field (30). These mechanisms ensure content is protected and conflicts are resolved efficiently, avoiding the complexity of different judicial systems and minimizing cross-border dispute risks (35).

However, while these platform-based enforcement mechanisms are essential, their implementation must be balanced against key legal safeguards and practical limitations. Automated content identification systems, though efficient, are prone to “false positives” that can wrongfully penalize legitimate content. Critically, these systems must be designed to account for principles such as fair use (in the U.S.) or fair dealing (in other jurisdictions), which permit the unauthorized use of copyrighted material for purposes like commentary, criticism, parody, and news reporting—activities that are central to the eSports content ecosystem.

Consequently, a robust enforcement strategy must be paired with transparent, accessible, and efficient counter-notice and appeals processes. These mechanisms are vital for ensuring due process, protecting transformative creator content, and preventing the overreach of rights holders, thereby maintaining a healthy balance between intellectual property protection and creative expression.

Revisiting the Palworld case, it is necessary to emphasize the importance of Trade Dress in eSports for brand recognition and fan loyalty. In a saturated market, a coherent visual identity allows fans to connect emotionally with organizations (56). A well-defined Trade Dress can evoke emotions and differentiate a team, but protecting it is challenging due to the rapid dissemination of digital content. A proposed approach should include developing a visual identity manual for video games. This manual would articulate the game's essence: its narrative, characters, aesthetic, color palette, and other unique attributes.

To operationalize this proposal, the visual identity manual should be conceived as a detailed implementation blueprint. Its scope must clearly delineate core brand assets, including logo usage, color codes (e.g., Pantone, HEX), typography, character poses, and the overall aesthetic of broadcast overlays, while also providing explicit rules for third-party use in community tournaments or streams.

We propose that this manual be published on a dedicated, publicly accessible website or developer portal, ensuring broad visibility and legal notice. Significantly, it should include a versioning system to adapt to game updates or brand evolutions. In a legal context, this publicly documented manual would serve as powerful evidence in establishing the distinctiveness of the trade dress and demonstrating a consistent effort to define and police the brand's identity, thereby strengthening infringement claims by undercutting any defense based on ambiguity or ignorance.

This approach is not merely theoretical; it mirrors well-established brand management strategies implemented by leading organizations in both traditional sports and digital media. For example, major leagues like the National Basketball Association (NBA) and global entities such as the Olympic Committee maintain extensive visual identity guidelines to ensure brand consistency across all platforms and merchandise. Within the gaming sector itself, developers like Riot Games (*League of Legends*) and Blizzard Entertainment (*Overwatch*) have successfully implemented similar frameworks.

They provide comprehensive asset packs and clear branding rules for third-party tournament organizers, content creators, and partners, thereby protecting their intellectual property while simultaneously empowering the community to engage with their brands in a controlled and consistent manner. These precedents demonstrate the viability and effectiveness of using a publicly articulated visual manual to manage a brand's identity at scale.

Strategically publishing this manual serves a dual preventive function. First, it publicly establishes what constitutes an authorized representation, making it difficult for third parties to claim ignorance. Second, a public manual strengthens brand identity, fostering quicker recognition from the audience and making it harder for imitations to be confused with the original. A comprehensive IP strategy is fundamental to the industry's sustainability and integrity (19).

However, a strategy centered on rigorous IP enforcement must be strategically balanced with a “community-aware” approach that recognizes the symbiotic relationship between rights holders and fans. The eSports ecosystem thrives on user-generated content, such as fan art, live streams, and community-run tournaments, which often exist in a legal gray area but are vital for a game's cultural relevance and longevity.

An overly aggressive enforcement policy risks alienating this passionate user base, potentially causing reputational damage that undermines the very brand value the IP is meant to protect. Therefore, a sustainable IP strategy, aligned with the principles of good governance, must distinguish between unauthorized commercial exploitation and non-commercial, transformative community engagement. This can be achieved by incorporating clear safe-harbor policies within the proposed visual identity manual, actively promoting a culture of respectful creativity, and viewing the fan community not as a threat, but as an important partner in brand stewardship.

A salient illustration of this tension is found in the enforcement actions taken by Nintendo. The company has frequently utilized cease-and-desist letters to shut down prominent, non-commercial fan projects, including the popular *Another Metroid 2 Remake* and *Pokémon Uranium*. While these enforcement actions are well within the company's legal rights, they consistently provoke significant community backlash. This response is often rooted in a perception that such legal maneuvers stifle the very creativity and passion that define the fan community, transforming non-commercial “labors of love” into legal liabilities and causing tangible reputational harm to the rights holder.

Similarly, Take-Two Interactive, parent company of Rockstar Games, faced a widespread community revolt after taking legal action against popular modding tools for *Grand Theft Auto V*. The modding community, which is integral to the game's longevity and cultural persistence, organized a massive “review bombing” campaign on the Steam platform, driving the game's recent reviews to “Overwhelmingly Negative”. This forced the company to publicly clarify its policy. These incidents serve as cautionary tales, demonstrating that the fan community is not a passive consumer but an active stakeholder whose negative reaction can inflict immediate and material damage on a brand, thereby complicating a purely enforcement-centric IP strategy.

## 7 Discussion

The strategic management of intellectual property in eSports can be effectively framed within the “twin transformation” concept articulated by Glebova & Madsen, which emphasizes the synergy between digitalization and sustainability. Our analysis suggests that a forward-thinking IP strategy is not merely a legalistic exercise but a core component of this transformation. Digitalization, represented by platform-based enforcement and data-driven monitoring, must be pursued in tandem with sustainability, which in this context translates to a stable, transparent, and fair governance ecosystem.

The proactive measures we propose—such as alternative dispute resolution, clear licensing frameworks, and the public visual identity manual—align with this model by fostering a predictable legal environment that supports long-term growth, stakeholder trust, and responsible community engagement, thus ensuring the industry's sustainable development.

The rapid maturation of the eSports industry has brought forth a complex legal landscape where intellectual property is no longer a secondary consideration but a central strategic element. The findings of this analysis underscore that intellectual property serves a dual role: it is both a protective shield for creative assets and a foundational pillar that drives innovation, competition, and economic sustainability within this dynamic sector. This reality necessitates a shift in perspective from purely reactive legal defense to proactive and comprehensive strategic management for all stakeholders involved.

The legal controversy surrounding the game *Palworld* serves as a critical contemporary case study, illustrating the precarious balance between creative inspiration and outright infringement. While drawing inspiration from existing works is a common practice in all creative fields, this case highlights the significant legal and reputational risks that arise when a new product's aesthetic and mechanics hew too closely to those of an established and iconic franchise. The ensuing debate moves beyond simple copyright of code or character design into more nuanced legal territory.

Specifically, the *Palworld* case brings the concept of Trade Dress to the forefront of the discussion in the digital entertainment space. Trade Dress, defined as the total image and overall impression of a product that identifies its source to the consumer, is a vital asset in eSports. In a market saturated with visual content, the distinct look and feel of a game, a team's branding, or an event's broadcast presentation are what build brand identity and foster a loyal fanbase.

The legal challenge lies in applying this doctrine, traditionally used for physical products and packaging, to the intangible and rapidly evolving virtual world of video games. The potential for consumer confusion, a key factor in Trade Dress disputes, is magnified in a digital environment where imitation can be quickly and widely disseminated. This situation underscores the urgent need for judicial systems to develop clearer precedents for protecting the overall aesthetic and sensory experience of a digital product against imitation.

To navigate this intricate environment, the importance of meticulously drafted contracts and license agreements cannot be overstated. These legal instruments form the bedrock of the intellectual property protection chain, providing essential clarity regarding the ownership, use, and distribution of creative assets. For eSports organizations, ensuring that all engagements with players, content creators, sponsors, and broadcast partners are governed by detailed agreements is a fundamental risk mitigation strategy that prevents future conflicts.

Beyond traditional legal tools, this paper's proposal of a publicly accessible visual identity manual presents an innovative and proactive strategy. Such a document would serve a dual purpose. First, it establishes a clear and public record of a brand's unique Trade Dress, making it difficult for infringers to claim ignorance. Second, it strengthens the brand's identity in the eyes of the community, fostering quicker recognition and making imitations less likely to be mistaken for the original work.

The digital platforms on which the eSports industry is built, such as Twitch and YouTube, also play a essential, if complex, role. Their automated content identification systems and established takedown procedures are indispensable tools for copyright holders. Fostering a collaborative relationship with these platforms is therefore a determining factor in the effective protection of creative works, allowing organizations to respond swiftly to infringements and minimize their impact.

Given the inherently global and cross-jurisdictional nature of eSports, reliance on national courts alone is often inefficient. The findings highlight the value of alternative dispute resolution mechanisms, particularly those offered by the World Intellectual Property Organization's Arbitration and Mediation Center. These forums provide a specialized and impartial path to resolving complex copyright and intellectual property disputes efficiently, thereby avoiding the costs and complexities of multi-jurisdictional litigation.

Ultimately, the integrity and continued growth of the eSports industry depend on a culture of respect for intellectual property, supported by a robust and multifaceted legal framework. The convergence of copyright, trademark, contract law, and the protection of Trade Dress creates a synergistic shield for creative and commercial assets. A comprehensive strategy that integrates these elements is not just a legal formality but an essential business practice that fosters a secure environment for investment, collaboration, and sustainable development.

### 7.1 Methodological implications

This study employs a qualitative, doctrinal legal research methodology to critically analyze the application of intellectual property law within the eSports ecosystem. The approach is primarily conceptual, moving beyond a mere description of existing legal frameworks to interrogate their theoretical underpinnings and practical limitations in a rapidly evolving digital context. The research synthesizes primary sources, such as case law and statutes, with a comprehensive review of secondary legal scholarship to construct a novel line of argumentation regarding the tensions between established IP doctrines and the unique challenges posed by eSports.

This methodological choice is deliberate, standing in contrast to empirical legal studies which might, for example, seek to quantify the prevalence of consumer confusion or measure the economic impact of IP infringements through surveys or data analysis. While such empirical approaches offer valuable insights into the law's real-world effects, they are not equipped to address the study's primary objective.

The central research problem herein is fundamentally conceptual and interpretive: it concerns the coherence, applicability, and theoretical limitations of existing legal doctrines when applied to a novel technological domain. Consequently, a doctrinal methodology is singularly pertinent as it facilitates a rigorous interrogation of the legal principles themselves, allowing for the critical analysis of case law, statutes, and legal theory required to navigate the tensions at the heart of the Palworld controversy.

The analytical process began with a systematic literature review to map the current scholarly and legal landscape. Sources were selected from high-impact databases, including HeinOnline, Scopus, and Web of Science, based on their thematic relevance and academic rigor. However, the core of the methodology was not the collection of these sources, but their critical evaluation. Rather than merely summarizing existing viewpoints, this study contrasts competing legal interpretations and challenges prevailing assumptions in the literature.

In this context, a doctrinal-hermeneutic approach was used to dissect key legal texts and judicial decisions, while a conceptual analysis served to deconstruct and question how intellectual property frameworks are being adapted—or failing to adapt—to the realities of the eSports industry. This critical engagement with the material provides the foundation for the paper's central arguments.

### 7.2 Concluding remarks

In recent years, the advancement of the Internet and improvements in video game quality have fueled exponential growth in their popularity. The rise of eSports has placed video games at the forefront of media and entertainment. Consequently, increased activity has heightened the probability of legal conflicts, making it beneficial for all actors to understand the legal issues at stake.

Intellectual property is a guiding principle in the eSports industry. Without a clear and robust legal framework, the sector would lack the rules for sustainable growth. The responsibility falls on legal teams of eSports organizations to design specific and detailed copyright license agreements. These agreements not only protect all parties but also foster collaboration and legal clarity.

The analyzed case, which presents legal uncertainty, implies the need for clearer legal frameworks. Such frameworks would become particularly relevant if a tribunal were to determine that a title indeed leverages the Trade Dress of another, potentially limiting the use of freedom of expression as a defense in such contexts. This hypothetical scenario is where intellectual property plays a key role.

First, copyrights form the protective foundation for original creations in eSports, including code, characters, music, and broadcasts. Securing ownership and properly managing these rights allows publishers and teams to control the reproduction and distribution of their works. A clear copyright strategy is the groundwork for preventing unauthorized exploitation.

Furthermore, contracts and licenses regulate relationships and the exploitation of intellectual property. Agreements between developers, teams, players, and sponsors must precisely define rights, limitations, and responsibilities. With clear and detailed licenses, the industry will have solid tools to avoid ambiguities and potential litigation, fostering a transparent and secure collaborative environment.

Trademark protection focuses on the distinctive signs that identify eSports teams, leagues, and events. Registering names, logos, and slogans grants holders exclusive rights to use them and prevent third parties from using similar signs that could cause consumer confusion. A solid trademark strategy helps build and protect the reputation and commercial value of eSports organizations.

Finally, the protection of Trade Dress covers the total appearance of a product or service. In eSports, this can apply to the visual presentation of events, team aesthetics, and broadcast identity. Protecting Trade Dress helps differentiate offerings in the market and prevents competitors from using similar presentations that could mislead consumers.

In summary, a comprehensive intellectual property strategy in eSports must consider the synergy between copyrights, contracts, licenses, and the protection of trademarks and Trade Dress. By coordinating these legal aspects, organizations can strengthen their legal management, deter infringements, protect their assets, and foster a fair and respectful competitive environment.
